# Herpes zoster burden in a private healthcare setting of Argentina

**DOI:** 10.3389/fpubh.2026.1748267

**Published:** 2026-05-29

**Authors:** Delfina Recart, Paula Scibona, Diego Caruso, Laura Jorge, Lucas Perelli, Marisa Sánchez, Astrid Smud, Jorge A. Gómez

**Affiliations:** 1 Sección de Farmacología Clínica, Hospital Italiano de Buenos Aires, Buenos Aires, Argentina; 2 Servicio de Clínica Médica, Hospital Italiano de Buenos Aires, Buenos Aires, Argentina; 3 GSK, Buenos Aires, Argentina; 4 Seccion Infectología, Servicio de Clínica Médica, Hospital Italiano de Buenos Aires, Buenos Aires, Argentina

**Keywords:** Argentina, burden, herpes zoster, incidence, postherpetic neuralgia

## Abstract

**Background:**

Herpes zoster (HZ) is a frequent condition that can lead to debilitating pain, with complications that may persist for months. The most frequent complication is postherpetic neuralgia (PHN), but ocular, nervous, and dermatological complications are also common. While the incidence of HZ increases with age, few studies have assessed its burden in Latin America, particularly in Argentina.

**Methods:**

We conducted a retrospective cohort study using the electronic health records of the Italian Hospital of Buenos Aires (HIBA) Health Plan from 2010 to 2022. We included members of the HIBA Health Insurance Plan aged over 18 years with HZ in the analysis. We calculated the incidence of HZ and its complications in the general population.

**Results:**

During the study period, an average of 138,977 members were followed up yearly, 8,122 cases of HZ were recorded, representing a mean incidence of 4.50 (95% confidence interval [CI]: 4.15–4.85) per 1,000 person-years (PY). The incidence increased with age, being lowest in adults 18–29 years (1.87 cases per 1,000 PY) and highest in adults 80–89 years (7.27 cases per 1,000 PY). PHN frequency was 50.85 cases per 1,000 HZ patients (95% CI: 37.31–64.39). The median duration of PHN was 981 days (interquartile range [IQR]: 487–1,718). Other complications included cutaneous (31.3 cases/1,000 HZ cases), ophthalmic (6.0 cases/1,000 HZ cases), and neurological (1.85 cases/1,000 HZ cases) complications. Recurrent HZ occurred in 4.25% of cases, with a median time to recurrence of 815 days (IQR: 308–1,890).

**Conclusion:**

These results illustrate the high burden of HZ and its complications in Argentina, highlighting the need for HZ prevention.

## 1 Introduction

Herpes zoster (HZ) is a painful syndrome resulting from the reactivation of the varicella-zoster virus (VZV), which remains dormant in the nervous system after the initial chickenpox episode resolves (1). The mechanisms leading to the virus’s reactivation are not completely understood, but an age-related decrease in cell-mediated immunity likely plays an important role (2). Every adult has an estimated one-in-three chance of developing HZ during their lifetime, and HZ incidence increases with age (3). Individuals with immunosuppression due to disease or immunosuppressive medications face an increased risk of developing HZ (4, 5). Comorbidities such as diabetes mellitus, cardiovascular disease, chronic obstructive pulmonary disease (COPD), human immunodeficiency virus (HIV), chronic kidney disease, chronic lung disease, and depression are also associated with an increased risk of HZ (6).

HZ can lead to complications, such as ocular, dermatological, and neurological (5). Postherpetic neuralgia (PHN), a persistent pain potentially lasting for months, is the most common complication of HZ, occurring in about 20% of HZ cases, although prevalence may vary depending on the definition and duration of pain considered (7, 8). In a study of patients with HZ and subsequent PHN aged ≥65 years, the mean duration of pain reported by patients was 3.3 years (standard deviation [SD] 4.0) (9), with duration ranging from 3 months to more than 10 years. HZ and its complications such as PHN impose a significant healthcare resource and economic burden on patients, healthcare systems, and employers and may have a substantial impact on patients’ health-related quality of life (10–14).

The incidence of HZ varies globally and differs between regions, with lower rates in European populations and with the highest rates in Asia (15). In Latin America, HZ incidence is less studied (16). A recent systematic literature review of HZ incidence in Latin America in high-risk populations reported 6.4–36.5 HZ cases per 1,000 person-years (PY) (17). However, no studies reported the disease burden in the general population.

Before Argentina introduced varicella vaccination in the universal mass vaccination program in 2015 (i.e., as universal varicella vaccination [UVV]), the seroprevalence of VZV in adults was estimated at 98.5% (18), indicating that most adults are currently at risk of developing HZ following VZV reactivation. A retrospective analysis of medical records in adults aged >60 years with HZ at the Italian Hospital of Buenos Aires (HIBA), Argentina, conducted between June 2013 and May 2014, found an incidence of 5.5 cases per 1,000 patients (19). However, recent evidence on the burden of HZ in Argentina is lacking.

This study aimed to describe the burden of HZ and its complications in individuals affiliated with the Health Plan of the HIBA.

## 2 Methods

### 2.1 Study design

This observational, descriptive, retrospective cohort study utilized electronic health records (EHR) from the HIBA Health Insurance Plan from January 1, 2010, to December 31, 2022. The primary objective was to estimate the frequency of HZ in HIBA Health Insurance Plan members aged ≥18 years. Secondary objectives included estimating the frequency of acute and subacute herpetic neuralgia, PHN, and other HZ complications (neurological, cutaneous, ocular, and others) by age and gender. The study also aimed to assess the recurrence rate of HZ by gender and age.

### 2.2 Participants

We identified and included all HIBA Health Insurance Plan members aged ≥18 years diagnosed with HZ between January 1, 2010, and December 31, 2022. Individuals were considered affiliates for the entire month in which they paid for the Health Insurance Plan. We included all HIBA Health Insurance Plan members who met the selection criteria during the period of interest, so no sampling method was used. HZ cases in patients were defined using the root term “Zoster” registered in the EHR and/or a positive polymerase chain reaction (PCR) result for HZ, and/or positive HZ antigen detection during the study period. We chose a root-term search methodology approach, developed and tested during an initial feasibility assessment, to accommodate the broad nature of the database clinical coding system. This ensured that all clinical entries containing the term “zoster” were captured, accounting for variations in terminology and ensuring that no relevant cases were omitted due to inconsistent data entry across different departments or clinicians. As this methodology could also potentially identify cases of primary varicella infection, such cases were excluded unless there was an associated claim for acyclovir.

### 2.3 Data source and setting

Argentina’s health system includes the private, public, and social security sectors. The HIBA Health Insurance Plan is a private health insurance system that offers comprehensive medical and health services to about 140,000 affiliates, representative of the metropolitan population of the Autonomous City of Buenos Aires in terms of demographic and socioeconomic characteristics.

The linked EHR databases recorded healthcare resource use by patients affiliated with the HIBA Health Insurance Plan. This included outpatient visits, hospitalizations, inpatient and outpatient medications, and all studies, evaluations, and procedures conducted at the institution.

### 2.4 Variables and definitions

Patients with HZ were identified as those patients with the root term “zoster” recorded in the EHR and/or a positive PCR result for HZ, and/or the presence of HZ antigen as described above. Hospital codes used for HZ complications in the secondary objectives and corresponding International Classification of Diseases, Tenth Revision (ICD-10) codes are provided in Supplementary Table 1.

Patient age was calculated based on the index date for inclusion purposes only (i.e., patients with HZ were included only if they were ≥18 years). Age-specific incidence considered the patient age on the first day of the month.

The HZ diagnosis date was the date of diagnosis record entry in the EHR or the date of PCR result entry if no diagnosis was recorded.

Pain duration was based on its onset and end. Onset was defined based on the date of prescription of anti-neuropathic medications commonly used to manage PHN; gabapentin, pregabalin, amitriptyline, valproic acid, lamotrigine, duloxetine, venlafaxine, capsaicin cream, lidocaine patches, imipramine, nortriptyline, desipramine, carbamazepine, oxcarbazepine, or tramadol. The end of pain was based on the date of the last dispensing (pharmacy withdrawal) of the related drug. We subclassified pain as acute herpetic neuralgia (pain during the first 2 weeks from the date of HZ diagnosis), subacute herpetic neuralgia (pain from 14 to 90 days after the date of HZ diagnosis), and PHN (pain from 90 days after the date of HZ diagnosis).

Recurrence was defined as (1) a new record added to the previously recorded HZ event after 90 days; (2) a new record of the root term “zoster” in the EHR, and/or a positive PCR result for HZ, and/or the presence of HZ antigen, after 90 days without HZ; or (3) a new record of the root term “zoster” in the EHR plus dispensing of related drug after 90 days without HZ.

Complications associated with HZ were classified as cutaneous, neurological, ocular, or other. Cutaneous complications considered superimposed infection with prescription of antibiotics—amoxicillin, amoxicillin-sulbactam, amoxicillin-clavulanate, trimethoprim-sulfamethoxazole, clindamycin, cephalexin—recorded in the EHR within 1 week of the HZ diagnosis. Non-PHN neurological complications were defined as hospitalization within 1 month of the HZ diagnosis with cerebrospinal fluid samples positive for HZ, including encephalitis, meningitis, and myelitis. Ocular complications were ophthalmic zoster with an ophthalmology consultation within 2 weeks of the HZ diagnosis. Other complications included conditions requiring a consultation with an ear, nose, and throat specialist within 2 weeks of the HZ diagnosis (including facial paralysis, tinnitus, and vertigo).

### 2.5 Statistical methods

We described categorical variables as absolute frequencies and proportions. We summarized continuous variables as mean and SD or median and interquartile range (IQR), according to the distribution of the data. The Shapiro-Wilks test assessed normality. The STATA statistical package (version 18, Stata Corp LLC, College Station, TX, United States) was used for the analysis.

To calculate the incidence of HZ, we divided the number of new cases of HZ during a specific period by the population at risk during that period, i.e., all patients in the HIBA Health Insurance Plan who had not experienced HZ at the start of the study. The population was estimated using the monthly number of patients in the HIBA Health Insurance Plan. We averaged the monthly incidence to obtain the annual estimate. The incidence of HZ was expressed as the number of new HZ cases per 1,000 individuals at risk per unit of time (month or year) for each age group and gender.

We calculated the proportion of HZ cases with acute herpetic neuralgia, subacute herpetic neuralgia, and PHN in HIBA Health Insurance Plan members per year and during the 2010–2022 period. The number of new cases was divided by the number of HIBA Health Insurance Plan members ≥18 years with HZ per month during the same period. PHN proportions were expressed per 1,000 HZ cases. For acute herpetic neuralgia, subacute herpetic neuralgia and PHN, the mean duration per case was calculated as the time in days between the onset of pain (defined as the date of first claim for analgesic in the pharmacy) and the cessation of pain (considered to be 30 days after the last analgesic claim from the pharmacy).

We calculated the proportion of HZ patients that develop PHN and other non-PHN complications in patients with at least 6 months of follow-up over 2010–2022. We used the number of identified HZ patients who developed complications as the numerator and the total number of patients diagnosed with HZ as the denominator, with frequency expressed as a percentage.

When feasible, we also stratified incidence, proportions, and durations by age groups and gender.

### 2.6 Ethical conduct of the study

This study complied with all laws regarding patient privacy. There was no direct patient contact or primary data collection at the individual level. We extracted anonymous data retrospectively from medical records. To preserve participant confidentiality, only those directly involved in the study had access to the collected data. This was done in accordance with Law 25,326 on the Protection of Personal Data of Argentina, Resolution 1480/11 of the Ministry of Health of Argentina, and Law No. 3301/09, Law on Protection of Rights of Subjects in Research in Health. It also complied with Resolution No. 595/MSGC/14, which outlines requirements and procedures for behavioral, socio-anthropological, and epidemiological research projects in the Autonomous City of Buenos Aires. This study was evaluated by the Ethics Committee of the Italian Hospital of Buenos Aires assigned by PRIISA.BA (approval number: 9841).

## 3 Results

### 3.1 Study population

During the 13 years of follow-up, an average of 138,977 individuals per year were evaluated, comprising 83,311 women and 55,666 men. Across the study period there was a general increase in number of persons affiliated with HIBA; with a total of 124,597 observation years in 2010 rising to 152,169 in 2022. Over the entire period, the study included a total of 1,806,698 PY of observation time, with 1,083,044 PY for females and 723,654 PY for males. Approximately 60% of the observed study years involved individuals aged ≥50 years.

The study population was a general population and chronic comorbidities were not routinely characterized, although select comorbidities were assessed for some years (Supplementary Table 2). In 2019, of the 144,541 individuals affiliated with HIBA, 12,239 (8.46%) had pre-existing diabetes mellitus, 5,371 (3.72%) had chronic obstructive pulmonary disease (COPD), 6,026 (4.17%) had chronic heart failure, and 581 (0.40%) were HIV-positive. In 2022 (152,169 affiliates) the relative prevalence of these conditions was diabetes mellitus (7.32%), COPD (2.91%), heart failure (2.81%), and HIV-positive individuals (0.37%).

### 3.2 Incidence of HZ

**Figure 1:**
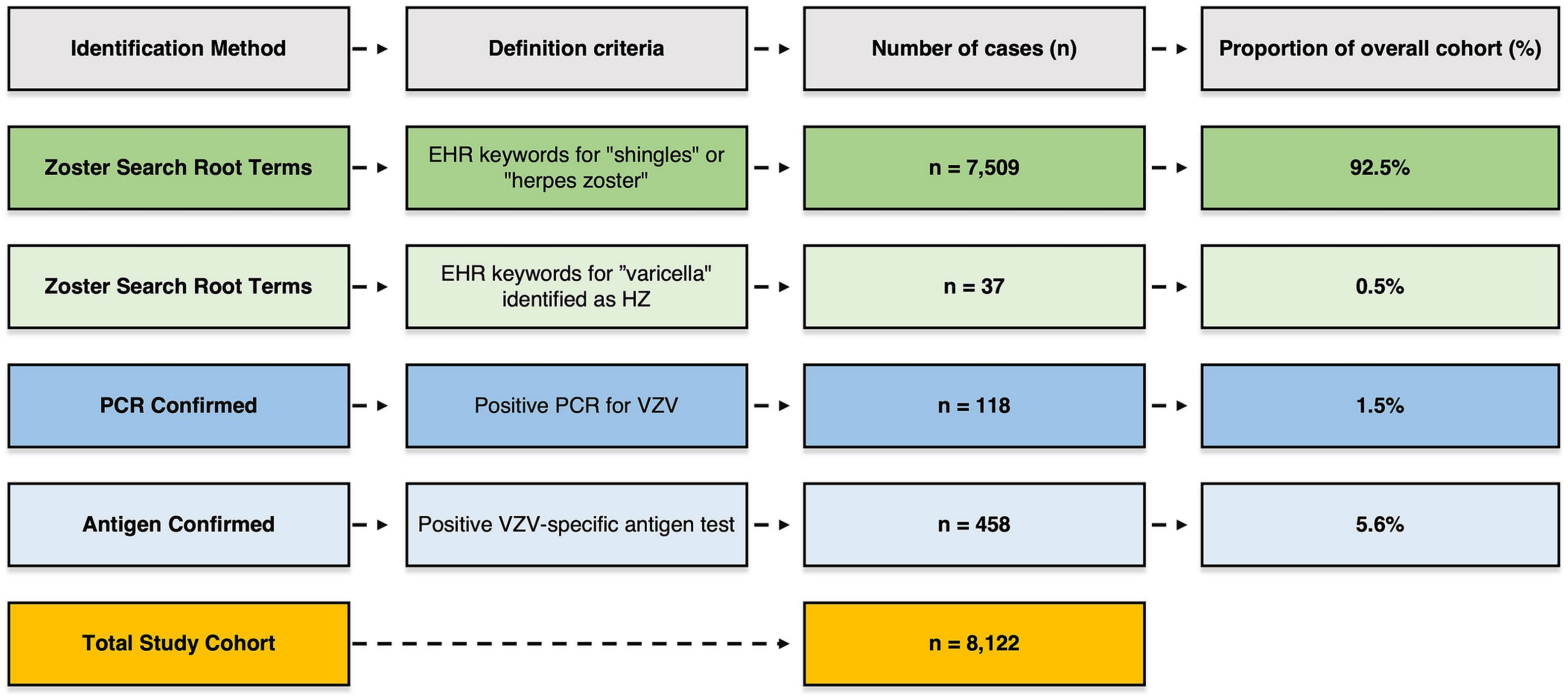
Flow chart highlighting HZ case identification. Note that percentages do not sum to 100% due to rounding. EHR, electronic health record; HZ, herpes zoster; PCR, polymerase chain reaction; VZV, varicella-zoster virus.

During the study period, 8,122 cases of HZ were recorded based on the defined search terms and laboratory diagnosis criteria (Figure 1). The great majority of cases (7,546, 92.9%) were diagnosed clinically and identified through codes within the EHR; this included 37 cases identified using the root term “varicella”, with none excluded, as all had an associated claim for acyclovir. For those cases identified through laboratory investigation criteria, 118 cases (1.5%) were identified through confirmatory PCR testing and 458 (5.6%) through HZ antigen testing.

The median age of patients with HZ was 68 years (IQR 55–78 years). Of these cases, 5,220 (64.27%) were women and 2,902 (35.73%) were men. The majority of HZ cases were in older adults, with 6,542 cases (80.55%) in individuals aged ≥ 50 years, and with 3,845 (47.34% of all cases) involving those aged ≥ 70 years. The analysis of HZ by age strata excluded 4 patients aged 100 years or above, resulting in 8,118 HZ cases for that analysis.

**Figure 2:**
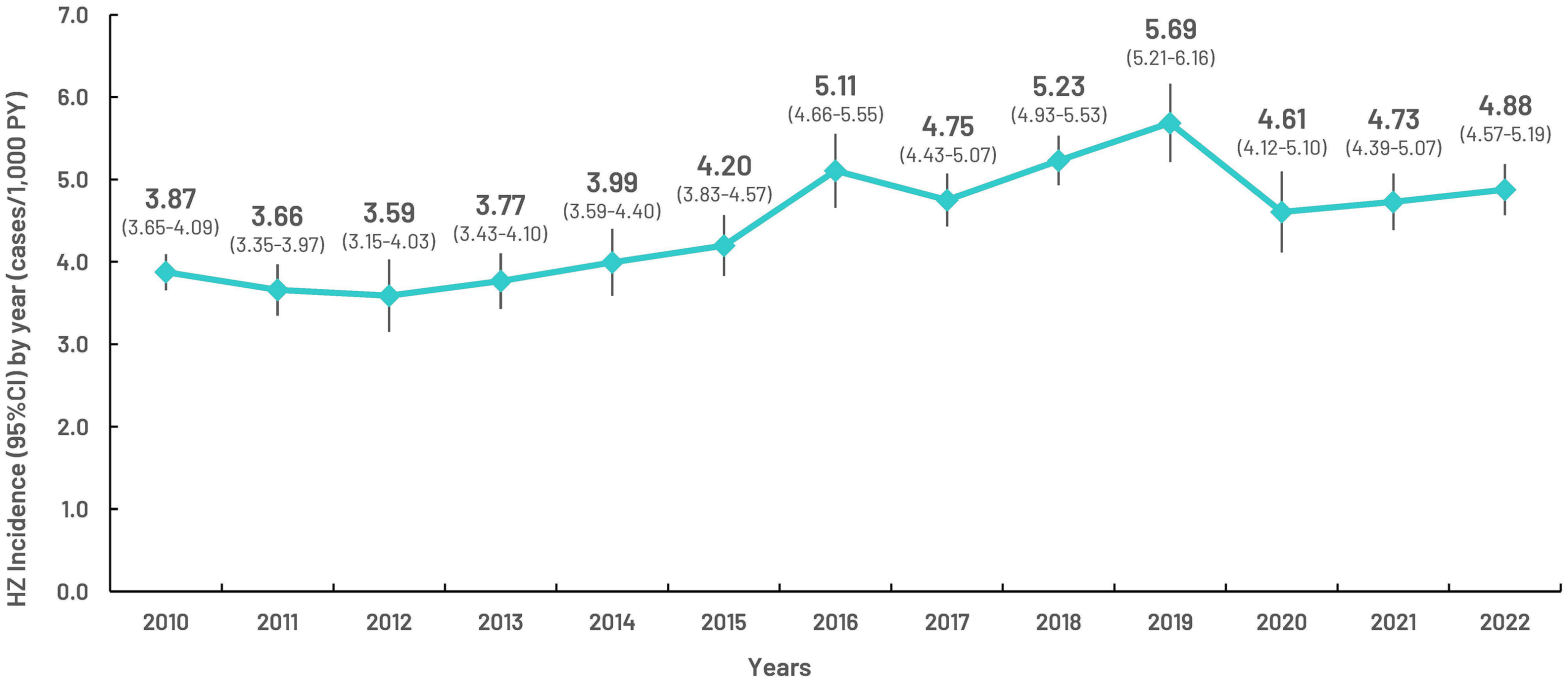
Annual incidence of HZ (per 1,000 PY). HZ, herpes zoster; CI, confidence interval; PY, person-years.

**Figure 3:**
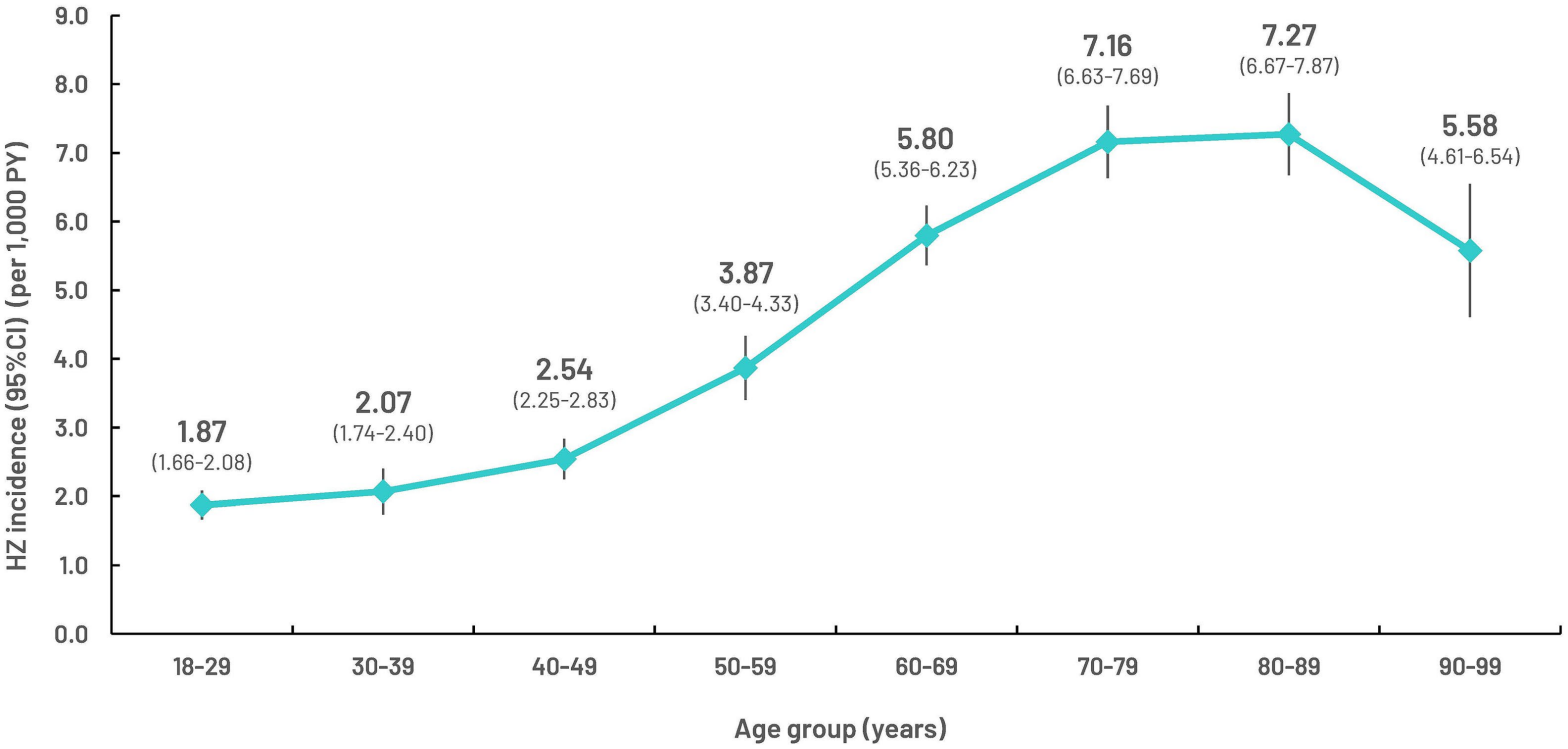
Mean incidence of HZ by age group (per 1,000 PY), 2010–2022. HZ, herpes zoster; PY, person-years.

The overall incidence of HZ (including recurrent cases) among members of the HIBA Health Insurance Plan was 4.50 per 1,000 PY (95% confidence interval [CI]: 4.15–4.85). HZ was more common in females, with an incidence of 4.82 per 1,000 PY (95% CI: 4.40–5.24) in women and 4.01 per 1,000 PY in men (95% CI: 3.74–4.28) (Table 1). The observed annual HZ incidence varied over the entire study period, ranging between 3.59 cases per 1,000 PY in 2012 to 5.69 cases per 1,000 PY in 2019, with some decline in 2020–22 (Table 1; Figure 2). The HZ incidence was lowest in the younger age group (18 to 29 years) with 1.87 cases per 1,000 PY (95% CI: 1.66–2.08) and highest in the 80 to 89 years age group with 7.27 cases per 1,000 PY (95% CI: 6.67–7.87) (Table 2; Figure 3).

**Table 1:** Annual incidence of HZ by gender, 2010–2022.

Year	Mean male population observed per year	Mean female population observed per year	Mean total population observed per year	HZ cases in males	HZ cases in females	Total HZ cases	HZ incidence per 1,000 men-year	HZ incidence per 1,000 women-year	HZ incidence per 1,000 PY
2010	49,867.2	74,729.8	124,597.0	171	312	483	3.43	4.18	3.88
2011	50,239.3	76,736.3	126,975.5	186	279	465	3.70	3.64	3.66
2012	51,922.9	79,419.8	131,342.8	164	307	471	3.16	3.87	3.59
2013	52,675.9	80,586.8	133,262.8	177	325	502	3.36	4.03	3.77
2014	53,351.8	81,324.3	134,676.1	198	340	538	3.71	4.18	3.99
2015	54,523.3	83,041.9	137,565.2	209	369	578	3.83	4.44	4.20
2016	55,643.5	84,218.5	139,862.0	248	466	714	4.46	5.53	5.11
2017	56,350.2	84,931.1	141,281.3	236	435	671	4.19	5.12	4.75
2018	57,557.5	86,078.8	143,636.3	279	472	751	4.85	5.48	5.23
2019	58,151.7	86,389.7	144,541.3	262	560	822	4.51	6.48	5.69
2020	59,087.4	86,833.2	145,920.6	228	444	672	3.86	5.11	4.61
2021	61,667.2	89,201.3	150,868.5	253	460	713	4.10	5.16	4.73
2022	62,616.2	89,552.6	152,168.8	291	451	742	4.65	5.04	4.88
Total period 2010–2022	**723,653.8**	**1,083,044.1**	**1,806,697.9**	**2,902**	**5,220**	**8,122**	**4.01**	**4.82**	**4.50**

HZ, herpes zoster; PY, person-years.

**Table 2:** Annual incidence of HZ by age, 2010–2022.

Age (years)	2010	2011	2012	2013	2014	2015	2016	2017	2018	2019	2020	2021	2022	Mean HZ incidence per 1,000 PY	SD	±95% CI
18–29	1.71	1.44	1.44	1.73	2.28	1.67	2.05	1.85	2.07	2.56	1.43	1.58	2.53	1.87	0.38	0.21
30–39	1.71	1.36	1.13	1.30	2.00	1.51	2.29	1.99	2.63	2.96	2.47	2.99	2.58	2.07	0.61	0.33
40–49	1.51	1.80	2.49	2.19	2.35	1.96	2.68	2.61	3.16	3.37	3.11	2.84	2.94	2.54	0.54	0.29
50–59	3.83	2.92	2.66	3.66	2.85	3.24	4.49	3.47	5.67	4.32	5.15	3.98	4.02	3.87	0.86	0.46
60–69	5.71	5.32	5.04	3.97	5.33	5.83	6.32	6.21	6.38	7.35	6.16	6.45	5.28	5.80	0.80	0.44
70–79	5.93	6.09	5.87	7.15	6.22	7.18	8.07	7.85	6.82	8.99	6.72	7.71	8.47	7.16	0.97	0.53
80–89	7.30	6.65	6.59	6.40	5.54	6.91	8.62	7.69	9.26	9.02	6.20	6.52	7.83	7.27	1.10	0.60
90–99	4.92	6.46	2.63	2.66	9.76	5.40	6.92	6.70	5.21	5.33	4.37	6.04	6.09	5.58	1.78	0.97
Total	3.88	3.66	3.59	3.77	3.99	4.20	5.11	4.75	5.23	5.69	4.61	4.73	4.88	4.47	0.64	0.35

CI, confidence interval; HZ, herpes zoster; PY, person-year; SD, standard deviation.

### 3.3 PHN and other complications

Table 3 presents the proportion of HZ cases with PHN by year and age during the study period. Among all individuals diagnosed with HZ, a PHN proportion of 50.85 cases per 1,000 HZ patients (95% CI: 37.31–64.39) was observed. The frequency of PHN varied by age, ranging from 21.74 PHN cases per 1,000 HZ cases in individuals aged 18–29 years to 65.50 PHN cases per 1,000 HZ cases in those aged 70–74 years. Frequency was higher in women (57.66 PHN cases/1,000 HZ patients; 95% CI: 42.36–72.97) than in men (38.59 PHN cases per 1,000 HZ patients; 95% CI: 27.27–49.92) (Table 4).

**Table 3:** Annual proportion of PHN per 1,000 patients with HZ by year and by age.

	Total HZ	Total PHN	PHN proportion (per 1,000 HZ cases)
By year			
2010	483	0	0
2011	465	1	2.15
2012	471	8	16.99
2013	502	19	37.85
2014	538	26	48.33
2015	578	41	70.93
2016	714	42	58.82
2017	671	47	70.04
2018	751	55	73.24
2019	822	57	69.34
2020	672	44	65.48
2021	713	40	56.10
2022	742	33	44.47
By age group (years)			
18–29	414	9	21.74
30–39	506	27	53.36
40–49	660	23	34.85
50–54	427	20	46.84
55–59	523	25	47.80
60–64	763	46	60.29
65–69	984	58	58.94
70–74	1,084	71	65.50
75–79	1,015	54	53.20
80–84	902	44	48.78
85–89	597	24	40.20
90–99	247	12	48.58
Overall	8,122	413	50.85

HZ, herpes zoster; PHN, postherpetic neuralgia.

**Table 4:** Annual proportion of PHN per 1,000 patients with HZ.

	HZ cases	PHN cases	PHN proportion^a^	SD	±95% CI	PHN proportion lower limit	PHN proportion upper limit
Females	5,220	301	57.7	24.9	13.5	42.4	73.0
Males	2,902	112	38.6	20.8	11.3	27.3	49.9
Total	8,122	413	50.8	28.2	15.3	37.3	64.4

a PHN proportion is expressed per 1,000 HZ cases.

CI, confidence interval; HZ, herpes zoster; PHN, postherpetic neuralgia; SD, standard deviation.

The median duration of PHN was 981 days (IQR, 487–1,718); the mean duration was 1,178.54 days (SD 861.78).

Among the 8,122 patients with HZ, 319 (3.93%) presented a complication other than PHN. Cutaneous complications (bacterial superinfection) were most frequently observed, affecting 254 patients (31.3 cases per 1,000 HZ cases). Ophthalmic complications were reported in 49 patients (6.0 cases per 1,000 HZ cases). Other complications were infrequently identified. Non-PHN neurological complications were observed in 15 HZ patients (1.85 cases per 1,000 HZ cases), and only 1 patient (0.12 cases per 1,000 HZ cases) had an otorhinolaryngological complication. The frequency of complications was highest in older individuals aged ≥80 years.

During the study period, 345 of all 8,122 HZ cases (4.25%) were identified as recurrent. Recurrences were slightly more frequent in women, accounting for 4.71% of HZ cases in women, compared to 3.41% of HZ cases in men. Recurrence rates increased marginally with age, notably above 55 years (Supplementary Table 3). The median time to recurrence was 815 days (IQR: 308–1,890). We did not observe an age-related pattern in this median time to recurrence (Supplementary Table 3).

## 4 Discussion

This study evaluated the burden of HZ and its complications in health plan members of the HIBA in Argentina for the period 2010–2022. During this period, 8,122 cases of HZ were recorded, with a mean incidence of 4.50 per 1,000 PY (95% CI 4.14–4.85). This aligns with previous global estimates. A systematic review found that the incidence rate of HZ ranged between 3 and 5 per 1,000 PY in North America, Europe, and Asia-Pacific (16), while another has reported an HZ incidence rate in Europe of 2.0–4.6 per 1,000 PY (20). Higher rates are reported elsewhere, e.g., an administrative claims data analysis from the United States has reported crude incidence rates in 2019 of 6.75 per 1,000 PY (21).

As in other studies across diverse geographical populations, we found HZ incidence to be higher in women than in men (6, 15, 21–31). The underlying biological reasons for this remain unclear (15, 22). Health-seeking behavior with greater consultation rates for health issues has also been proposed, although there is limited objective evidence for this, with some studies concluding greater risk in females remains after adjustment for consultation rates (22, 30). We would note that in the present study, while the overall pattern was of higher HZ incidence in females, and that this was seen for all study years (except 2011), there was no consistent gender disparity across all age-strata (i.e., in some years for some age-strata, the HZ incidence was higher in men than in females). HZ incidence rates also increased with age, again consistent with the findings of numerous previous studies (6, 15, 20, 21, 23–32), where age-related decline in VZV-specific cell-mediated immunity contributes to VZV re-activation (33). The highest incidence rate of HZ in our study was seen in the age group 80 to 89 years, with a rate of 7.27 HZ cases per 1,000 PY.

A general increase in HZ incidence over the study period was observed, which is consistent with previous reports evaluating temporal trends in HZ incidence (15, 26). Several explanations have been proposed for this trend. The exogenous booster hypothesis suggests that rising incidence could be linked to childhood varicella vaccination programs, which reduce exposure to wild-type VZV and limit immune boosting through re-exposure (34). Other perspectives attribute the general increase in HZ incidence in most countries to demographic changes (with greater life expectancy leading to a gradual shift to an increase in the older population), greater use of immunosuppressants, and improved health literacy and awareness of HZ, which may lead to more frequent consultation and diagnosis (35–37). In Argentina a childhood UVV program has been in place since 2015, with significant reductions in varicella incidence both in the target childhood population (aged 1–4 years) and in the broader population, although to a lesser extent (38). Although our study was not designed to formally evaluate any potential impact of UVV on HZ incidence, incidence rates over 2010–2015 ranged from 3.88–4.20 per 1,000 PY, while rates in subsequent years were higher and greatest in 2019 (5.69 per 1,000 PY) immediately prior to the coronavirus disease 2019 (COVID-19) pandemic. While one could speculate that this higher incidence in the period subsequent to the introduction of UVV reflects an impact of reduced exogenous boosting, epidemiologic data from other countries with established varicella immunization programs indicate that increasing HZ incidence rates were already apparent prior to UVV introduction, and that there has been no notable change in the magnitude of rate increases following introduction (39–41). To some extent our results reflect similar patterns. As such, while we observed an increase after 2015, we would be cautious about drawing any conclusions as to any impact of varicella vaccination on the HZ incidence trends observed in our study population.

We also found a decline in HZ incidence during 2020–2022 from the peak observed in 2019. This could be due to a number of reasons. This may reflect a shift in health-seeking behaviors and some element of under-detection during the COVID-19 pandemic period. Although data are limited, other administrative claims-based studies elsewhere have also reported declines in HZ during this same period compared with 2019 pre-pandemic rates, including studies from the United States reporting reduced HZ incidence across most age-strata (21), and also a Spanish study reporting lower HZ hospitalization rates (42). However, other recent data from Japan has shown that while there was a marginal decline in incidence rates in 2020, this was then followed by higher incidence, beyond that observed in the immediate pre-pandemic period (31). It should be noted that COVID-19 infection itself may contribute to immune dysregulation and greater risk of VZV reactivation, with studies reporting greater HZ risk in patients with COVID-19 (43–45). Some data also points towards a potential association between COVID-19 vaccines and HZ risk (45–47), although data are inconsistent on this (48, 49). In this context, one may anticipate potentially higher HZ incidence in 2020–2022, which is contradictory to that observed in the study from the United States reporting on this period (21), and also as found in the present study, which may indicate some element of under-reporting. We would note that in the present study, while there was no apparent change in the frequency of non-PHN complications (presenting in the acute disease phase) in the 2020–2022 period compared with the preceding years, there was some decline in the observed frequency of PHN in 2020 and 2021, which may lend some support to change in health-seeking behavior and reduced consultations during this time as an explanation for these reduced HZ incidence rates 2020–2022 we report. However, this remains uncertain.

Among cases of HZ, the proportion of PHN was 50.85 per 1,000 patients. A recent systematic literature review of the incidence of HZ complications estimated PHN incidence at 26–467 per 1,000 individuals in the general population (5). In our study, PHN was more frequent in women (57.66 PHN cases per 1,000 HZ patients) than in men (38.59 PHN cases per 1,000 HZ patients). Higher consultation rates among women in the health plan population may explain this gender difference, or it could denote a real difference in PHN burden between genders. Data are inconsistent for this, with some studies reporting a higher prevalence of PHN in females (23, 25, 50–53) although other analyses have shown no such association (54, 55). Differences in psychosocial stressors and negative life events may also contribute to increased PHN rates in women (56). As with HZ incidence, prior literature shows that risk of PHN development and PHN rates (whether incidence rates, or frequency as a proportion of HZ cases as we report) increases with age (5, 21, 23, 25, 31, 50–53, 57–59). The highest proportion of PHN in our study was observed in the 70 to 74 years age group (with rates of 65.50 PHN cases per 1,000 HZ cases), then declined in older age groups. The PHN rates we report are somewhat lower than those reported from other countries, where approximately 5–30% of patients with HZ develop PHN (16). In addition, data from other countries (e.g., Spain and Italy) indicate that rates increase sharply after 50 years of age and peaks around 80 years of age (52, 60). In the present study differences in PHN rates in different age-strata were relatively marginal, and as stated above peaked in the 70–74 years age group, with lower rates observed in those aged ≥80 years.

Specific explanations for this are uncertain. We used anti-neuropathic pharmacy claims actioned by individuals more than 90 days after the date of HZ diagnosis as a proxy for diagnosis of PHN, with the date of the last prescription filled serving to indicate resolution and allow estimation of PHN duration. Use of medication pharmacy claims is an established method to quantify the proportion of patients with HZ who develop PHN (23, 51, 61, 62). However, this approach may be subject to some bias, both in relation to PHN identification, and its use to evaluate duration of PHN pain (where it serves as a measure of treatment duration as a proxy for duration of pain). Possible explanations for this fall-off in PHN rates in the oldest age-strata may include lower consultation rates or underdiagnosis among older adults. In addition, medications may also be obtained through non-study pharmacies or otherwise obtained outside the hospital network and would not be captured in the HIBA database. Furthermore, patients may discontinue medication due to side effects, cost, or ineffectiveness rather than pain resolution, which may also underestimate PHN detection, and also pain duration.

From a temporal perspective, in the early study years (2010–2015), the PHN proportion increased from 0 in 2010 to 70.93 per 1,000 HZ patients in 2015 before plateauing from 2015 to 2020, and then declining in 2021–2022. In addition to the points made above, this lower prevalence of PHN in the early study period can partly be attributed to an initial underreporting of medication claims used to identify PHN cases at the onset of the EHR system and that, in the early years of the study, patients were able to obtain medication outside the hospital pharmacy, potentially leading to incomplete records in the hospital’s database. As regards the decline in PHN frequency in 2022, we would note that the PHN proportion was calculated using all HZ cases for that year as a denominator. However, that will underestimate the PHN proportion (as HZ cases occurring in the last 3 months (i.e., Q4) of that year cannot be included in PHN case numbers). A post-hoc adjustment (excluding all HZ cases from Q4 in 2022) shows an adjusted proportion of 61.91 per 1,000. Similar aspects relate to the 2010 data, although there, the impact would be less. The mean PHN duration in our study was 1,178.5 days (i.e., 3.2 years), closely mirroring the 3.3 years reported in a previous study from the United States in adults aged ≥65 years, where patients directly self-reported the existence of continued pain (9). Another French cohort study involving 108 patients with PHN who attended a chronic pain clinic (most aged ≥60) years reported a mean PHN duration of 22.7 months (SD, 28.1; median: 13.3 months) at the time of initial clinic consultation (63).

Among HZ patients, 3.93% developed complications other than PHN. The most frequent were cutaneous (3.13%), while the least frequent were otorhinolaryngological (0.01%). Ocular complications were rare in our study (0.60%), contrasting with a systematic literature review reporting an incidence of up to 4.36% (5). Indeed, in general, the frequency of all such complications was lower than that reported elsewhere (5). Estimates may vary due to different definitions used and included complications. For example, we used a rather limited definition for cutaneous complications (bacterial superinfection), while others would also include disseminated zoster in this category (5). Furthermore, the requirement for 6-months continuous enrollment following a HZ diagnosis when assessing complications may have contributed to underestimation. In addition, it is well recognized that database studies such as the present study are prone to underestimating true complication rates due to coding omissions (64).

In our study, 4.25% of HZ cases were recurrent, aligning with recurrence rates reported in the literature. A systematic review of HZ incidence worldwide noted recurrence rates ranging from 1 to 6%, and recurrence risk was 5 to 6% in studies with long-term follow-up (16).

This study presents the limitations and potential biases inherent to observational, descriptive, retrospective, and real-world evidence studies based on secondary databases. The analysis was susceptible to coding inaccuracies. HZ cases and complications may have been missed due to incorrect coding or classification, which may have led to a bias in the results. Some codes may have been used for exclusion purposes, rather than as actual diagnostic labels, potentially resulting in misclassification. To mitigate this, we combined multiple identification criteria in search strategies, including related cases, PCR, and/or antigen testing results, although such laboratory testing is not routinely used in clinical practice for HZ diagnosis. When assessing HZ complications, we included only patients with a 6-month follow-up, which may have underestimated complication incidence. As discussed above, we calculated pain duration based on filled medication claims. However, a dispensed medication does not necessarily indicate that the medication was taken as prescribed. Therefore, the treatment duration as calculated here may not accurately reflect the true disease duration. In addition, only dispensing information data from pharmacies affiliated with the HIBA Health Insurance Plan was captured in the study. Given that the Health Plan requires monthly fees, offers comprehensive coverage, and provides substantial medication discounts in affiliated pharmacies, the likelihood of members seeking care or purchasing medications elsewhere is low. We restricted HZ recurrence estimates to incident cases identified between 2010 and 2022, potentially missing cases or recurrences occurring outside of this period. Finally, results may not be generalizable to country-level estimates or other settings, as populations may differ in access to care, medical practice, and treatment guidelines. Therefore, these findings should be interpreted with caution.

To our knowledge, this is the largest epidemiological study conducted in Argentina regarding HZ and its complications. Having local data is essential to understand the true burden of the disease and to develop evidence-based prevention and treatment strategies.

## Data Availability

For requests for access to anonymised subject level data, please contact the corresponding author.
