# Supplementary material for: Herpes zoster burden in a private healthcare setting of Argentina
Source: Front Public Health. 2026 May 29;14:1748267. doi: 10.3389/fpubh.2026.1748267 (PMC13260107; doi:10.3389/fpubh.2026.1748267)
Supplement: Supplementary file 1 [file supplementary_file_1.docx]

Supplementary Material

# Supplementary Table 1. List of hospital codes used for herpes zoster and complications and equivalence with ICD-10 codes

| **Hospital Code** | **ICD-10 code** |
| --- | --- |
| Dorsal shingles | B02 Herpes zoster |
| Left frontal herpes zoster | B02 Herpes zoster |
| Herpes zoster | B02 Herpes zoster |
| Thoracic herpes zoster | B02 Herpes zoster |
| Herpes zoster ophthalmic | B02.3 Ocular herpes zoster |
| Intercostal herpes zoster | B02 Herpes zoster |
| Disseminated herpes zoster | B02.7 Disseminated herpes zoster |
| Shingles on the thigh | B02 Herpes zoster |
| Shingles on the arm | B02 Herpes zoster |
| Herpes zoster dermatitis on the face | B02 Herpes zoster |
| Cervical herpes zoster | B02 Herpes zoster |
| Herpes zoster pain | B02.8 Herpes zoster with other complications |
| Costal herpes zoster | B02 Herpes zoster |
| Trigeminal herpes zoster | B02.2 Herpes zoster with other nervous system involvement |
| Genital herpes zoster | B02 Herpes zoster |
| Abdominal herpes zoster | B02 Herpes zoster |
| Herpes zoster dermatitis | B02 Herpes zoster |
| Dorsal herpes zoster suspected | B02 Herpes zoster |
| Herpes zoster on the left lower limb | B02 Herpes zoster |
| Lumbar herpes zoster | B02 Herpes zoster |
| Multimetameric herpes zoster | B02.7 Disseminated herpes zoster |
| Oral herpes zoster | B02 Herpes zoster |
| Herpes zoster gluteal | B02 Herpes zoster |
| Herpes zoster with neuralgia | B02.2 Herpes zoster with other nervous system involvement |
| Suspect trigeminal herpes zoster | B02.2 Herpes zoster with other nervous system involvement |
| Suspected shingles on the arm | B02 Herpes zoster |
| Facial herpes zoster | B02 Herpes zoster |
| Shingles on the facial nerve | B02 Herpes zoster |
| Brachial zoster | B02 Herpes zoster |
| Herpes zoster on the right leg | B02 Herpes zoster |
| Herpes zoster in the frontal region | B02 Herpes zoster |
| Herpes zoster on the right side of the face | B02 Herpes zoster |
| Right dorsal herpes zoster | B02 Herpes zoster |
| Shingles on the head | B02 Herpes zoster |
| Shingles on the trunk | B02 Herpes zoster |
| Ophthalmic herpes zoster | B02.3 Ocular herpes zoster |
| Herpes zoster in left upper limb | B02 Herpes zoster |
| Shingles on the left leg | B02 Herpes zoster |
| Left lumbar herpes zoster | B02 Herpes zoster |
| Inguinal herpes zoster | B02 Herpes zoster |
| Herpes zoster on the left buttock | B02 Herpes zoster |
| Shingles on the right flank | B02 Herpes zoster |
| Herpes zoster on the right upper limb | B02 Herpes zoster |
| Herpes zoster on the lip | B02 Herpes zoster |
| Right intercostal herpes zoster | B02 Herpes zoster |
| Suspected herpes zoster | B02 Herpes zoster |
| Left facial and ocular herpes zoster | B02.3 Ocular herpes zoster |
| Right thoracic herpes zoster | B02 Herpes zoster |
| Herpes zoster on the shoulder | B02 Herpes zoster |
| Shingles on the left arm | B02 Herpes zoster |
| Left thoracic herpes zoster | B02 Herpes zoster |
| Shingles on the right sciatic nerve | B02.2 Herpes zoster with other nervous system involvement |
| Herpes zoster in the region right cervicothoracic | B02 Herpes zoster |
| Breast herpes zoster | B02 Herpes zoster |
| Herpes zoster keratitis | B02.8 Herpes zoster with other complications |
| Suspected thoracic herpes zoster | B02 Herpes zoster |
| Herpes zoster intergluteus | B02 Herpes zoster |
| Herpes zoster on the lower limb | B02 Herpes zoster |
| Shingles on the neck | B02 Herpes zoster |
| Herpes zoster in the twelfth segment of the dorsal spinal cord | B02.2 Herpes zoster with other nervous system involvement |
| Herpes zoster in the lumbar root | B02.2 Herpes zoster with other nervous system involvement |
| Herpes zoster on the right shoulder | B02 Herpes zoster |
| Nasal herpes zoster | B02 Herpes zoster |
| Suspected intercostal herpes zoster | B02 Herpes zoster |
| Herpes zoster on the scalp | B02 Herpes zoster |
| Herpes zoster in the right lower limb | B02 Herpes zoster |
| Left intercostal herpes zoster | B02 Herpes zoster |
| Herpes zoster on the leg | B02 Herpes zoster |
| Left dorsal herpes zoster | B02 Herpes zoster |
| Herpes zoster on the left upper limb | B02 Herpes zoster |
| Right lumbar herpes zoster | B02 Herpes zoster |
| Shingles on the left hand | B02 Herpes zoster |
| Suspected shingles on the neck | B02 Herpes zoster |
| Herpes zoster in the left brachial plexus | B02.2 Herpes zoster with other nervous system involvement |
| Herpes zoster in the right hemicranium | B02 Herpes zoster |
| Suspected abdominal herpes zoster | B02 Herpes zoster |
| Sacral herpes zoster | B02 Herpes zoster |
| Herpes zoster on the left shoulder | B02 Herpes zoster |
| Shingles on the left thigh | B02 Herpes zoster |
| Shingles on the right buttock | B02 Herpes zoster |
| Herpes zoster on the right thigh | B02 Herpes zoster |
| Herpes zoster on the right arm | B02 Herpes zoster |
| Lumboabdominal herpes zoster | B02 Herpes zoster |
| Right cervical herpes zoster | B02 Herpes zoster |
| Herpes zoster in region behind the ear | B02 Herpes zoster |
| Herpes zoster in the armpit | B02 Herpes zoster |
| Herpes zoster in the upper limb | B02 Herpes zoster |
| Shingles on both buttocks | B02 Herpes zoster |
| Herpes zoster on the left side of the face | B02 Herpes zoster |
| Herpes zoster in the ear | B02 Herpes zoster |
| Herpes zoster in the occipital region | B02 Herpes zoster |
| Suspected disseminated herpes zoster | B02.7 Disseminated herpes zoster |
| Right frontal herpes zoster | B02 Herpes zoster |
| Herpes zoster with postherpetic neuralgia | B02.8 Herpes zoster with other complications |
| Suspicion of herpes zoster on the right buttock | B02 Herpes zoster |
| Herpes zoster in the left inguinal region | B02 Herpes zoster |
| Herpes zoster keratitis in the right eye | B02.3 Ocular herpes zoster |
| Herpes zoster of the right ophthalmic nerve | B02.3 Ocular herpes zoster |
| Cervicobrachial herpes zoster | B02.2 Herpes zoster with other nervous system involvement |
| Herpes zoster in both lower limbs | B02 Herpes zoster |
| Suspected shingles on the thigh | B02 Herpes zoster |
| Herpes zoster encephalitis | B02 Encephalitis due to herpes zoster |
| Herpes zoster of the left ophthalmic nerve | B02.3 Ocular herpes zoster |
| Right upper dorsal herpes zoster | B02 Herpes zoster |
| Herpes zoster in the root of the third lumbar spinal nerve | B02.2 Herpes zoster with other nervous system involvement |
| Herpes zoster on the mandibular nerve | B02.2 Herpes zoster with other nervous system involvement |
| Shingles on the right hand | B02 Herpes zoster |
| Right ophthalmic herpes zoster | B02.3 Ocular herpes zoster |
| Acute abdominal herpes zoster | B02 Herpes zoster |
| Herpes zoster on the left flank | B02 Herpes zoster |
| Brachial herpes zoster | B02.2 Herpes zoster with other nervous system involvement |
| Lumbosacral herpes zoster | B02 Herpes zoster |
| Herpes zoster of the knee | B02 Herpes zoster |
| Suspected left facial herpes zoster | B02 Herpes zoster |
| Herpes zoster on the right browbone | B02.3 Ocular herpes zoster |
| Herpes zoster on the eyelid | B02.3 Ocular herpes zoster |
| Herpes zoster in the left groin | B02 Herpes zoster |
| Facial zoster | B02 Herpes zoster |
| Right trigeminal herpes zoster | B02.2 Herpes zoster with other nervous system involvement |
| Progressive outer retinal necrosis due to herpes zoster virus | B02.3 Ocular herpes zoster |
| Right intercostal herpes zoster of the seventh- eighth and ninth dorsal nerve | B02.2 Herpes zoster with other nervous system involvement |
| Meningitis due to herpes zoster | B02.1 Meningitis due to herpes zoster |
| Suspected zoster infection | B02 Herpes zoster |
| Herpes zoster infection in the right third dorsal metamere | B02.2 Herpes zoster with other nervous system involvement |
| Herpes zoster in the upper maxillary nerve | B02.2 Herpes zoster with other nervous system involvement |
| Superinfected herpes zoster | B02.8 Herpes zoster with other complications |
| Herpes zoster on the right breast | B02 Herpes zoster |
| Herpes zoster in the left eye | B02.3 Ocular herpes zoster |
| Herpes zoster in the first segment of the right dorsal spinal cord | B02.2 Herpes zoster with other nervous system involvement |
| Herpes zoster in the third segment of the lumbar spinal cord | B02.2 Herpes zoster with other nervous system involvement |
| Herpes zoster at the level of the second sacral vertebra | B02.2 Herpes zoster with other nervous system involvement |
| Herpes zoster dermatitis on the cheek | B02 Herpes zoster |
| Mild to moderate recurrent dorsal herpes zoster | B02 Herpes zoster |
| Shingles on the right hip | B02 Herpes zoster |
| Herpes zoster on both upper limbs | B02 Herpes zoster |
| Herpes zoster in the third segment of the right lumbar spinal cord | B02.2 Herpes zoster with other nervous system involvement |
| Herpes zoster in the left abdominal region | B02 Herpes zoster |

ICD-10: International Classification of Diseases, Tenth Revision.

# Supplementary Table 2. Select comorbidities* within the study population in 2019 and 2022

| **Year** | **Total population observed** | **Diabetes Mellitus**  **n (%)** | **COPD**  **n (%)** | **Heart failure**  **n (%)** | **HIV**  **n (%)** |  |
| --- | --- | --- | --- | --- | --- | --- |
|  |  |  |  |  |  |  |
| 2019 | 144,541 | 12,239 (8.46%) | 5,371 (3.72%) | 6,026 (4.17%) | 581 (0.40%) |  |
| 2022 | 152,169 | 11,145 (7.32%) | 4,424 (2.91%) | 4,270 (2.81%) | 566 (0.37%) |  |

*Comorbidities identified via relevant ICD-10 codes

COPD: chronic obstructive pulmonary disease, HIV: Human immunodeficiency virus infection; ICD-10: International Classification of Diseases, Tenth Revision.

# Supplementary Table 3. Frequency and time to HZ recurrence by age

| **Age group (years)** | **Number of HZ cases with recurrence** | **Total HZ cases number** | **Recurrence frequency (%)** | **Time to recurrence (median, Q1, Q3)** |
| --- | --- | --- | --- | --- |
| **18–29** | 13 | 414 | 3.1% | 1,317 (534–2,055) |
| **30–39** | 12 | 506 | 2.4% | 970 (439–1,780) |
| **40–49** | 22 | 660 | 3.3% | 690 (299–2,218) |
| **50–54** | 14 | 427 | 3.3% | 1,421 (777–3,238) |
| **55–59** | 23 | 523 | 4.4% | 480 (245–1,857) |
| **60–64** | 32 | 763 | 4.2% | 1,400 (557–3,494) |
| **65–69** | 49 | 984 | 5.0% | 971 (255–2,205) |
| **70–74** | 45 | 1,084 | 4.2% | 1,159 (532–2,143) |
| **75–79** | 43 | 1,015 | 4.2% | 987 (308–2,053) |
| **80–84** | 46 | 902 | 5.1% | 536 (192–1,633) |
| **85–89** | 34 | 597 | 5.7% | 462 (226–1,041) |
| **90–99** | 12 | 247 | 4.9% | 288 (190–371) |
| **Total** | **345** | **8,122** | **4.2%** | **815 (308–1,890)** |

HZ: herpes zoster; Q: quartile.
